# Interventions for promoting the initiation of breastfeeding

**DOI:** 10.1590/1516-3180.20141321T1

**Published:** 2014-02-01

**Authors:** Lisa Dyson, Felicia M. McCormick, Mary J. Renfrew

## Abstract

**BACKGROUND:**

Despite the widely documented health advantages of breastfeeding over formula feeding, initiation rates remain relatively low in many high-income countries, particularly among women in lower income groups.

**OBJECTIVE:**

: To evaluate the effectiveness of interventions which aim to encourage women to breastfeed in terms of changes in the number of women who start to breastfeed.

**METHODS:**

: *Search methods:* We searched the Cochrane Pregnancy and Childbirth Group's Trials Register (July 2007), handsearched the Journal of Human Lactation, Health Promotion International and Health Education Quarterly from inception to 15 August 2007, and scanned reference lists of all articles obtained. *Selection criteria:* Randomized controlled trials, with or without blinding, of any breastfeeding promotion intervention in any population group except women and infants with a specific health problem. *Data collection and analysis:* One review author independently extracted data and assessed trial quality, checked by a second author. We contacted investigators to obtain missing information.

**MAIN RESULTS:**

Main results: Eleven trials were included. Statistical analyses were conducted on data from eight trials (1553 women). Five studies (582 women) on low incomes in the USA with typically low breastfeeding rates showed breastfeeding education had a significant effect on increasing initiation rates compared to standard care (risk ratio (RR) 1.57, 95% confidence interval (CI) 1.15 to 2.15, P = 0.005). Subgroup analyses showed that one-to-one, needs-based, informal repeat education sessions and generic, formal antenatal education sessions are effective in terms of an increase in breastfeeding rates among women on low incomes regardless of ethnicity and feeding intention. Needs-based, informal peer support in the antenatal and postnatal periods was also shown to be effective in one study conducted among Latina women who were considering breastfeeding in the USA (RR 4.02, 95% CI 2.63 to 6.14, P < 0.00001).

**AUTHORS' CONCLUSIONS:**

This review showed that health education and peer support interventions can result in some improvements in the number of women beginning to breastfeed. Findings from these studies suggest that larger increases are likely to result from needs-based, informal repeat education sessions than more generic, formal antenatal sessions. These findings are based only on studies conducted in the USA, among women on low incomes with varied ethnicity and feeding intention, and this raises some questions regarding generalisability to other settings.

**This is the abstract of a Cochrane Review published in the Cochrane Database of Systematic Reviews (CDSR) 2005, issue 2, Art. No. CD001688. DOI:** 10.1002/14651858.CD001688.pub2 (http://cochrane.bvsalud.org/cochrane/main.php?lib=COC&searchExp=Interventions%20and%20for%20and%20promoting%20and%20the%20and%20initiation%20and%20of%20and%20breastfeeding〈=pt).

**The full text is available from:** http://onlinelibrary.wiley.com/doi/10.1002/14651858.CD001688.pub2/pdf.

**The abstract is available in the Portuguese, Spanish and Chinese languages from:** http://summaries.cochrane.org/pt/CD001688/intervencoes-para-encorajar-as-mulheres-a-iniciar-o-aleitamento-materno.

## COMMENTS 

The prevalence of exclusive breastfeeding until the sixth month of life has increased from 2.5% to 38.6% in Brazil over the last 20 years due to three key factors: NBCAL (the Brazilian rules for marketing of breastmilk substitutes), improvement of the mothers' socioeconomic levels and improvement of their educational profiles.

This interesting review demonstrates that antenatal education for mothers focusing on the benefits of breastfeeding and organization of groups of lactating mothers has a positive influence on improvement of the prevalence of breastfeeding. Thus, prenatal consultations for pregnant women, including with a pediatrician (as recommended by the Brazilian Society of Pediatrics), are effective actions that contribute towards improvement of the prevalence of breastfeeding. 

**Rubens Feferbaum.** Associate Professor of Pediatrics, School of Medicine, Universidade de São Paulo (USP), and Attending Physician in the Neonatal Intensive Care Unit, Children's Institute, Hospital das Clínicas, Universidade de São Paulo (HC/USP), São Paulo, Brazil.
